# Effect of Quorum Quenching Lactonase in Clinical Isolates of *Pseudomonas aeruginosa* and Comparison with Quorum Sensing Inhibitors

**DOI:** 10.3389/fmicb.2017.00227

**Published:** 2017-02-14

**Authors:** Assia Guendouze, Laure Plener, Janek Bzdrenga, Pauline Jacquet, Benjamin Rémy, Mikael Elias, Jean-Philippe Lavigne, David Daudé, Eric Chabrière

**Affiliations:** ^1^URMITE, Aix-Marseille Université UM63, CNRS 7278, IRD 198, INSERM 1095 IHU – Méditerranée InfectionMarseille, France; ^2^Laboratoire de Biologie Moléculaire et Cellulaire, Université des frères Mentouri ConstantineConstantine, Algérie; ^3^Gene&GreenTKMarseille, France; ^4^Department of Biochemistry, Molecular Biology and Biophysics and Biotechnology Institute, University of Minnesota, St. PaulMN, USA; ^5^INSERM, U1047, University of Montpellier 1Montpellier, France; ^6^Department of Microbiology, Caremeau University HospitalNîmes, France

**Keywords:** quorum quenching, quorum sensing, lactonase, pyocyanin, bacterial virulence, biofilm, proteases, anti-bacterial agents

## Abstract

*Pseudomonas aeruginosa* is a Gram negative pathogenic bacterium involved in many human infections including otitis, keratitis, pneumonia, and diabetic foot ulcers. *P. aeruginosa* uses a communication system, referred to as quorum sensing (QS), to adopt a group behavior by synchronizing the expression of certain genes. Among the regulated traits, secretion of proteases or siderophores, motility and biofilm formation are mainly involved in the pathogenicity. Many efforts have been dedicated to the development of quorum sensing inhibitors (QSI) and quorum quenching (QQ) agents to disrupt QS. QQ enzymes have been particularly considered as they may act in a catalytic way without entering the cell. Here we focus on the lactonase *Sso*Pox which was previously investigated for its ability to degrade the signaling molecules, acyl-homoserine lactones, in particular on the engineered variant *Sso*Pox-W263I. We highlight the potential of *Sso*Pox-W263I to inhibit the virulence of 51 clinical *P. aeruginosa* isolates from diabetic foot ulcers by decreasing the secretion of two virulence factors, proteases and pyocyanin, as well as biofilm formation. We further compared the effect of *Sso*Pox-W263I to the comprehensively described QSI, 5-fluorouracil and C-30. We found the lactonase *Sso*Pox-W263I to be significantly more effective than the tested QSI at their respective concentration optimum and to retain its activity after immobilization steps, paving the way for future therapeutic applications.

## Introduction

*Pseudomonas aeruginosa* is a human opportunistic pathogen involved in many infection types and which causes serious health complications (Stover et al., 2000; Driscoll et al., 2012). In 2006/2007, this Gram negative bacterium alone was responsible for 8% of general healthcare associated infections in the USA (Sievert et al., 2013). *P. aeruginosa* is involved in both community-acquired and hospital-acquired infections including otitis, keratitis, wound and burn infections, pneumonia and urinary tract infections (Driscoll et al., 2012). Furthermore, *P. aeruginosa* is, along with *Staphylococcus aureus*, the most common pathogen isolated from diabetic foot infections notably in South East Asia, and is also highly involved in infections when an alteration of the skin occurs (Driscoll et al., 2012).

Many bacteria, including *P. aeruginosa*, use a molecular communication system, referred to as quorum sensing (QS), to synchronize the expression of certain genes and adopt a group behavior. Among QS-regulated traits, virulence factors production such as pyocyanin and proteases (Hentzer, 2003), motility (Daniels et al., 2004) and biofilm formation (Whiteley et al., 2001) are involved in the development of infections.

Quorum sensing in *P. aeruginosa* depends on four different hierarchically organized systems: Las, Iqs, Rhl and Pqs. The first system to be activated is the Las system which depends on the production and perception of an acyl-homoserine lactone (AHL): *N-*(3-oxododecanoyl)-homoserine lactone (OdDHL or 3-oxo-C_12_ AHL; Gambello and Iglewski, 1991; Pearson et al., 1994). Induction of the Las system triggers the expression of the Las protease and elastase and activates the other QS systems. Despite its dominant role in the QS circuitry, eliminating LasR activation only results in a delayed activation of the *Pseudomonas* quinolone signal (PQS) system but does not completely eliminate QS (Diggle et al., 2003). In addition, the QS system of *P. aeruginosa* is interconnected with other regulatory networks involved in environmental cues such as phosphate, iron and oxygen sensing (Lee and Zhang, 2014).

Regarding the importance of bacterial communication in the development of virulence, strategies for QS disruption, known as quorum quenching (QQ), have emerged to maintain bacteria in a commensal lifestyle. To this end, quorum sensing inhibitors (QSI) and QQ enzymes have been particularly considered (Dong et al., 2007; Kalia, 2013; Tang and Zhang, 2014; Brackman and Coenye, 2015; Fetzner, 2015). QSI, such as brominated furanones, aim to prevent bacteria from perceiving endogenous QS molecules. Pyrimidine analog has also been reported as a QS disruptor (Ueda et al., 2009). QQ enzymes such as acylases or lactonases degrade AHL signals (Bzdrenga et al., 2016; Rémy et al., 2016a). Among these, the enzyme *Sso*Pox, isolated from the archaea *Sulfolobus solfataricus*, has been considered based on both its lactonase activity and tremendous stability due to its extremophile origin (Rémy et al., 2016b).

Many QQ examples have been reported and describe the reduction of virulence factor secretion (e.g., siderophores, proteases, rhamnolipids, etc.; Diggle et al., 2007; Welsh and Blackwell, 2016) and/or biofilm formation, especially using QSI molecules (Hentzer, 2003). However, a large proportion of these reports are dedicated to investigating model strains *P. aeruginosa* PAO1 and PA14, and only a few reports have described the response of clinical isolates to QS disruption, whereas natural isolates frequently harbor mutations in QS genes (Ciofu et al., 2010).

In this article, we investigated the effectiveness of the QQ enzyme *Sso*Pox-W263I, a variant of *Sso*Pox with increased catalytic effectiveness against 3-oxo-C_12_ AHL (Hiblot et al., 2013; Rémy et al., 2016b), previously reported as being efficient to drastically reduce the mortality in a rat pneumonia model (Hraiech et al., 2014), to modulate virulence factors in 51 clinical *P. aeruginosa* isolates collected from diabetic foot ulcers. We also compared its QQ potential to the most common QSI, the brominated furanone C-30 and the pyrimidine analog 5-fluorouracil (5-FU), by quantifying three virulence factors: pyocyanin production, protease secretion and biofilm formation (Ren et al., 2001; Ueda et al., 2009). Finally the lactonase was immobilized to assess its ability to functionalise medical devices and was proved to maintain sufficient activity for QQ.

## Materials and Methods

### Bacterial Strains and Growth Conditions

Experiments were performed with *P. aeruginosa* strains from samples held by the Department of Microbiology of the Nîmes University Hospital. The strains were isolated from diabetic patients with a suspected newly presenting episode of diabetic foot infection for a period of 1 year (2014). All the patients received an oral information, were anonymized and gave a non-opposition statement to bacterial storage. This study was approved by the local ethics committee (South Mediterranean III) and was carried out in accordance with the Declaration of Helsinki as revised in 2008. The samples were frozen at -80°C. Bacterial strains were cultivated on Luria Bertani (LB) agar plates at 37°C.

The model strains *P. aeruginosa* PAO1 and PA14 (Taxonomy ID: 208964 and 652611) and the clinical isolates were inoculated from a single colony and pre-cultivated in LB (10 g l^-1^ NaCl, 10 g l^-1^ tryptone, 5 g l^-1^ yeast extract) for 6 h at 37°C with shaking at 650 rpm. Subsequently, 3 ml of LB supplemented with 2% sheep blood (Biomérieux, France) was inoculated with 3 μl pre-culture and incubated at 37°C with shaking at 650 rpm. Pyocyanin production and protease activity were measured 24 h post-inoculation. Biofilm weight was determined 48 h post-inoculation.

The enzyme *Sso*Pox was added at 0.5 mg ml^-1^ and the QSI 5-FU and C-30 (Sigma) were used at 60 and 30 μM, respectively, as determined by a dose response experiment (Supplementary Figures 1–3).

### Bacterial Identification

All strains were checked using Matrix-assisted laser desorption ionization–time of flight (MALDI-TOF) as previously described (Seng et al., 2009). In brief, isolates were grown overnight on blood agar at 37°C under aerobic conditions. Single colonies were applied as a thin film to a 96-spot steel plate (BrukerDaltonics) and allowed to visibly dry at room temperature. Subsequently, 2 μl of MALDI matrix (saturated solution of alpha-cyano-4-hydroxy-cinnamic acid, 500 μl HPLC-grade acetonitrile, 475 μl HPLC-grade water, 25 μl trifluoroacetic acid) was applied on the spots and dried at room temperature. Isolates were tested in duplicate by MALDI-TOF mass spectrometry. The MALDI target plate was introduced into a microflex LT MALDI-TOF mass spectrometer for automated measurement and controlled by the FlexControl 3.3 (Bruker^®^) program. The spectra were collected in a mass range between 2,000–20,000 m/z then analyzed using the Bruker Biotyper 3.0 software package and compared to reference spectra for identification.

### Protein Production and Purification

Enzyme production was performed using *E. coli* BL21 (DE3)-pGro7/GroEL strain (TaKaRa) carrying plasmid pET22b-*Sso*Pox-W263I as previously described (Hiblot et al., 2012, 2013). In brief, cells were grown in ZYP medium supplemented with 100 μg ml^-1^ ampicillin and 34 μg ml^-1^ chloramphenicol at 37°C until OD_600nm_ reached 0.8–1. L-arabinose was added to a final concentration of 0.2% (w/v) in order to induce chaperones expression. Subsequently, 0.2 mM CoCl_2_ was added and the temperature was reduced to 23°C for an additional 20 h. Cells were harvested by centrifugation (4,400 *g*, 4°C, 20 min), the supernatant was discarded and the pellet was resuspended in lysis buffer [50 mM HEPES pH 8.0, 150 mM NaCl, 0.2 mM CoCl_2_, 0.25 mg ml^-1^ lysozyme, 0.1 mM Phenylmethylsulfonyl fluoride (PMSF) and 10 μg ml^-1^ DNaseI] and stored at -80°C overnight. Frozen cells were thawed at 37°C for 15 min and disrupted by three 30 s sonication steps (QSonica sonicator Q700; amplitude at 45). Cell debris was removed by centrifugation (21,000 *g*, 4°C, 15 min). Crude extract was incubated for 30 min at 80°C and was further centrifuged to precipitate *E. coli* proteins (21,000 *g*, 4°C, 30 min). *Sso*Pox-W263I was concentrated by overnight ammonium sulfate precipitation (75% saturation), and resuspended in activity buffer (50 mM HEPES pH 8.0, 150 mM NaCl, 0.2 mM CoCl_2_). Remaining ammonium sulfate was eliminated via desalting (HiPrep 26/10 desalting, GE Healthcare; ÄKTA Avant). The obtained protein sample was concentrated to 2 ml and subsequently loaded onto a size-exclusion chromatography column and purified to homogeneity (HiLoad 16/600 Superdex^TM^ 75pg, GE Healthcare; ÄKTA Avant). The purity of the protein was checked by 10% SDS-PAGE (Supplementary Figure 4) separation and protein concentration was measured using a NanoDrop 2000 spectrophotometer (Thermo Scientific).

### Proteolytic Activity

Cell-free culture supernatants were prepared by centrifugation for 5 min at 12,000 *g*. Protease activity was determined using azocasein (Sigma, St. Louis, MO, USA) as a substrate (Chessa et al., 2000). The reaction was performed in Phosphate-buffered saline (PBS) solution pH 7.0 with 50 μl of azocasein (30 mg ml^-1^ in water) and with 25 μl of culture supernatant for a final volume of 750 μl. The reaction was incubated at 37°C for 1 h and stopped by adding 125 μl of 20% (w/v) trichloroacetic acid. The blank assay was realized using 25 μl of culture medium with and without 0.5 mg ml^-1^ of *Sso*Pox. After centrifugation at 12,000 *g* for 5 min, the absorbance of the supernatant was measured at OD_366nm_ using a plate reader (Synergy HT, BioTek, USA).

### Pyocyanin Production

Pyocyanin was extracted from 500 μl of cell-free supernatant using 250 μl of chloroform. The mix was vortexed for 20 s, and centrifuged at 12,000 *g* for 5 min. The absorbance of the lower organic phase was measured at OD_690nm_ using a plate reader (Synergy HT, BioTek, USA; Price-Whelan et al., 2007).

### Biofilm Weight Measurement

After 48 h, each culture was sieved through a 100 μm pore-size cell strainer (Corning, New York, NY, USA) to separate biofilm from planktonic cells. The biofilm was washed with 2 ml PBS and centrifuged at 600 *g* for 5 min. Biofilms were weighed directly in the cell strainers using a precision scale (Supplementary Figure 5).

### Immobilization

In a 25 cm^2^ culture flask (Corning, New York, NY, USA), 1 ml of 5% Impranil^®^ DLU polyurethane (Covestro, Leverkusen, Germany) mixed with 20 mg ml^-1^ of *Sso*Pox-W263I and 0.5% Glutaraldehyde (Sigma) in purified water was dried over 12 h at 37°C. As a control, the same volume of activity buffer was added instead of *Sso*Pox-W263I. Before being used for culture, the flask was rinsed with 3 ml of purified water and then 3 ml of LB.

### Enzymatic Activity Measurement

Enzymatic activities were measured after 24 h based on the paraoxonase activity of *Sso*Pox using ethyl paraoxon as a substrate and the apparition of para-nitrophenol, paraoxon degradation product, was followed at OD_405nm_.

Released enzyme was measured after 24 h from culture supernatants. 5 μl of cell-free culture supernatant was transferred into a 96-well plate containing 95 μl of activity buffer (50 mM HEPES, 150 mM NaCl, pH 8). Then 100 μl of 2 mM ethyl paraoxon (Sigma) in activity buffer was added and OD_405nm_ was monitored during 10 min with a plate reader (Synergy HT, BioTek, USA) and the slope corresponding to paraoxon degradation kinetics was calculated.

Fixed enzyme was determined by adding 3 ml of a 1 mM ethyl paraoxon solution. After 3 min of incubation with shaking (300 rpm), 200 μl of paraoxon solution was transferred into a 96-well plate and OD_405nm_ was measured. The slope (OD min^-1^) was calculated and compared to a standard with a known enzyme concentration to calculate the amount of enzyme immobilized in the PU coating.

## Results

### Dose-Response Determination

*Pseudomonas aeruginosa* model strains PAO1 and PA14 were used to determine the optimal concentrations of the QQ enzyme *Sso*Pox and the QSI 5-FU and C-30 to decrease virulence factor production. Time points for sampling were determined by performing dose response experiments at different concentrations (Supplementary Figures 1–3). Under our conditions and in the absence of quenchers, protease and pyocyanin levels were similar for both strains (data not shown). The use of *Sso*Pox did not induce any delay in growth and maximal quenching was obtained for a concentration of 0.5 mg ml^-1^ of enzyme corresponding to 14.5 μM after 16 h of cultivation (Supplementary Figure 1). At this time point, pyocyanin production was almost completely abolished for both strains and protease production was reduced to 10% as compared to the control (untreated sample) for PAO1 and 43% for PA14. Biofilm formation was measured after 48 h to allow complete biofilm formation and direct weighing of cell aggregates (Supplementary Figure 5). The addition of *Sso*Pox (0.5 mg ml^-1^) reduced biofilm formation by 90% for PAO1 and 60% for PA14, and increasing *Sso*Pox concentration did not enhance quenching. The QSI molecule 5-FU only weakly impacted production of pyocyanin and protease in PAO1 but had a greater influence on PA14, with pyocyanin and protease productions reduced to 10 and 50%, respectively, compared to the untreated culture. Biofilm formation was reduced in both strains when concentrations of 5-FU of 60 μM or higher were used. According to these results, QQ of the clinical isolates was tested using 0.5 mg ml^-1^ *Sso*Pox or 60 μM 5-FU and 30 μM C-30. The same values were also reported in previous studies (García-Contreras et al., 2013, 2015). Pyocyanin and protease productions were determined after 24 h and biofilm formation 48 h post-inoculation.

Importantly, the addition of 5-FU to the culture led to a delay in growth, but this delay was recovered after 24 h for PAO1 (Supplementary Figure 6). The second QSI molecule, C-30, had much less impact in our experiment set-up as protease level remained up to 90% of the control for both PAO1 and PA14, and pyocyanin production was not reduced.

### Quenching of Virulence Factors by *Sso*Pox

**FIGURE 1:**
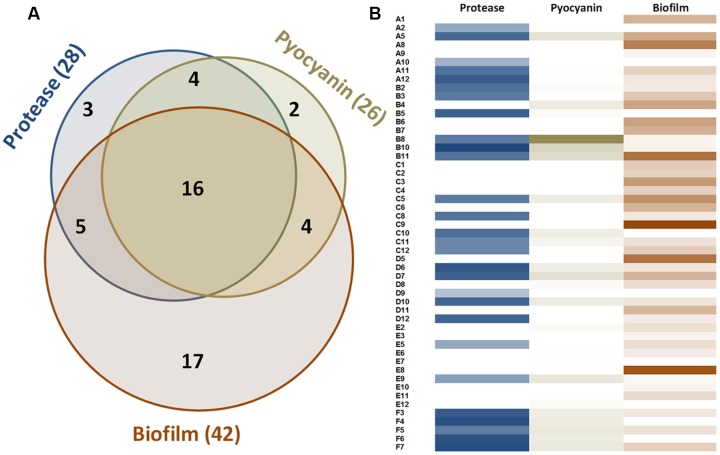
**Measurable virulence factor expression among the 51 clinical isolates of *P. aeruginosa*. (A)** Venn diagram describing the distribution of the 51 strains according to their production of virulence factors. **(B)** Proteolytic activities are presented according to OD_366nm_ value ranging from 0 to 1.37. Pyocyanin values represent the OD_690nm_ and range from 0 to 1. Biofilm values correspond to the weight of biofilm formed after 48 h for 3 ml of culture and range from 0 to 289 mg.

**FIGURE 2:**
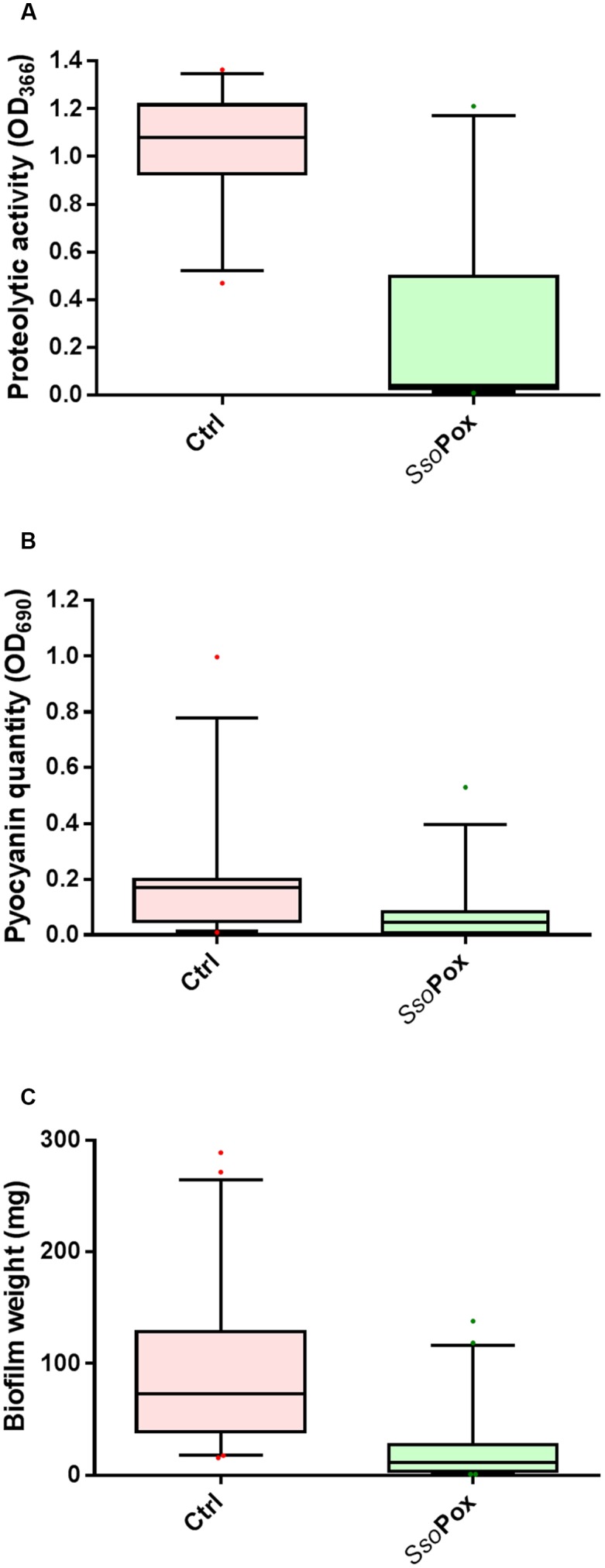
**Distribution plots of virulence factor values with and without quenching treatment of the 51 clinical isolates**. Values of proteolytic activity **(A)**, pyocyanin quantity **(B)** and biofilm formation **(C)** are shown as whiskers plots down to the 5th percentile and up to the 95th. Control samples (without enzyme) are shown in red while *Sso*Pox treated samples are in green. *Sso*Pox was added to cell cultures at a concentration of 0.5 mg ml^-1^. Points below and above the whiskers represent outlier values.

**FIGURE 3:**
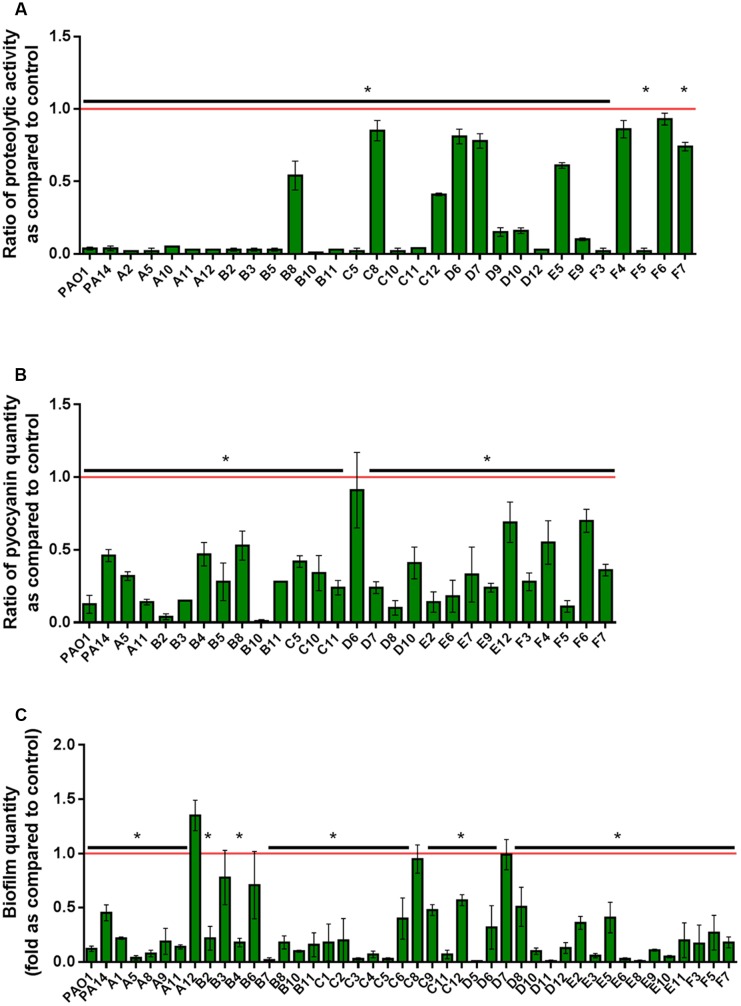
**Quenching of virulence factors using *Sso*Pox**. For each strain, bars represent the mean ratios of protease **(A)**, pyocyanin **(B)** and biofilm **(C)** levels between the treated culture with 0.5 mg ml^-1^ *Sso*Pox versus the untreated culture of three experiments. Error bars represent the standard deviations of three replicated experiments. ^∗^*p-values*<0.05 according to Student’s *t*-test.

In the experimental conditions used here, the two model strains PAO1 and PA14 both produced the three virulence factors tested. Among the 51 clinical isolates of *P. aeruginosa*, 16 strains produced the three virulence factors studied, 13 produced two virulence factors and 22 produced only one virulence factor (**Figure 1**). Proteolytic activities ranged from 0.47 to 1.37 with a median value at 1.08 (**Figure 2A**). Median pyocyanin production, among the 26 strains producing pyocyanin, was measured at 0.17 and this virulence factor showed the highest variations ranging from 0.01 to 1.00 (**Figure 2B**). Finally, the most common feature between the clinical isolates was biofilm formation as 42 strains out of 51 produced biofilm. Among these strains, the median value for wet biofilm was 73 mg with values ranging from 15 to 289 mg (**Figure 2C**). *Sso*Pox was used at a concentration of 0.5 mg ml^-1^ and pyocyanin and protease quantities were determined after 24 h. In these conditions, *Sso*Pox reduced protease activity of 26 out of 28 strains, with 20 of them being reduced by more than 50% and complete quenching was achieved for 16 of them. No clinical isolate harbored a higher value upon treatment with *Sso*Pox than without (**Figure 3A**). Pyocyanin production was reduced in 25 strains out of 26 and 20 of them were reduced by more than half the control level. Furthermore, pyocyanin production was completely eliminated in six strains. As noticed for protease production, no strain had increased pyocyanin production when the QQ enzyme *Sso*Pox was added (**Figure 3B**). Biofilm was reduced in 37 strains out of 42, 36 of them were reduced by more than half and 20 of them completely lost their ability to grow in biofilms. Unlike pyocyanin and protease production, one clinical isolate, A12, had a slightly thicker biofilm upon treatment with *Sso*Pox than without but this difference was not statistically significant (**Figure 3C**).

### Comparison between *Sso*Pox and the QSI 5-FU and C-30

**FIGURE 4:**
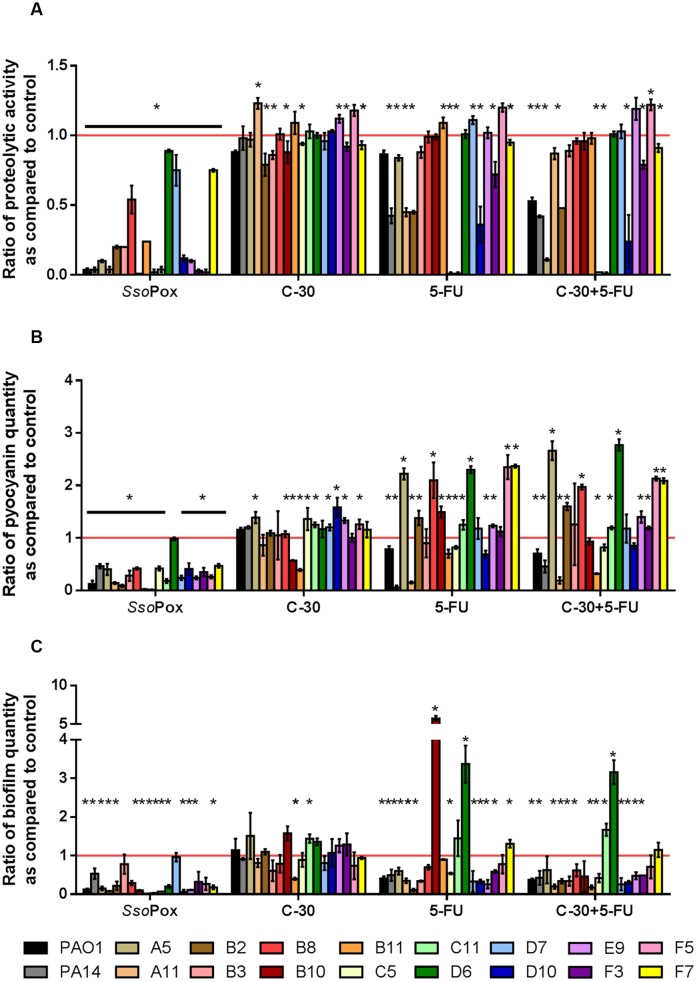
**Comparison of quenching by *Sso*Pox and QSI C-30 and 5-FU**. For each strain, bars represent the mean ratios of protease **(A)**, pyocyanin **(B)** and biofilm **(C)** levels between the treated culture with optimal concentrations of quorum quenching (QQ) agents (0.5 mg ml^-1^ *Sso*Pox, or 30 μM C-30 and/or 60 μM 5-FU) versus the untreated culture of three experiments. Error bars represent the standard deviations of three replicated experiments. ^∗^*p-values*<0.05 according to Student’s *t*-test.

To compare QQ potentials between *Sso*Pox and QSI molecules, the 16 clinical strains producing the three virulence factors were tested with 0.5 mg ml^-1^ *Sso*Pox, 30 μM C-30, 60 μM 5-FU and both QSI. Under our conditions, C-30 showed very little QQ potential either with the model strains PAO1 and PA14 or with the clinical isolates (**Figure 4** and Supplementary Figure 3). Only five strains showed a slight decrease in protease production, the maximum decrease measured was 20% for strain B2. Pyocyanin production was only reduced for two strains, B10 and B11, with ratios of 0.57 and 0.39, respectively. Concerning biofilm formation only one strain showed a statistically significant decrease with a ratio of 0.4 as compared to the control. More importantly, many strains showed significantly higher virulence factor production after treatment with C-30.

The results obtained using 5-FU indicated more QQ activity than C-30 in these experimental conditions. Indeed, seven strains out of 16 presented decreased proteolytic activity, two strains had decreased pyocyanin production and 10 strains formed less biofilm upon treatment with 5-FU. The combination of both QSI molecules did not lead to an increase in quenching potential as compared to the use of 5-FU alone. *Sso*Pox was the most active QQ agent, reducing proteolytic activities in every strain tested, reducing pyocyanin production in all but one strain and reducing biofilm formation in 12 strains out of 16.

### Immobilization

**FIGURE 5:**
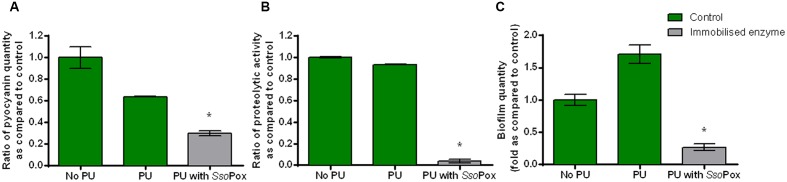
**Quenching *P. aeruginosa* PAO1 with enzyme immobilized in polyurethane (PU)**. Bars represent the mean ratios of pyocyanin **(A)**, protease **(B)** and biofilm **(C)** levels between the treated culture with immobilized *Sso*Pox (PU with *Sso*Pox) versus the untreated culture containing a PU coating or without coating (No PU). Error bars represent the standard deviations of two replicate experiments. ^∗^*p-values*<0.05 according to Student’s *t*-test using control with PU as reference.

To prove the effectiveness of *Sso*Pox-W263I in medical device-like conditions, the enzyme was immobilized in a waterborne polyurethane coating with the cross-linking agent glutaraldehyde. The influence of the PU coating without enzyme on growth and virulence factor production of PAO1 model strain was assessed to ensure that PU did not have a major impact on bacterial metabolism. Neither growth nor protease production differed. Pyocyanin and biofilm productions were slightly impacted with a decrease of pyocyanin production and an increase in biofilm formation. Despite these differences inherent to PU coating the impact of enzyme could be measured and quenching was successfully achieved (**Figure 5**). Indeed, in the same culture conditions as free enzymes, the immobilized *Sso*Pox was able to decrease pyocyanin production by 2.1, protease activity by 24 and biofilm quantity by 6.5 as compared to the control with polyurethane coating. Enzyme release was determined by measuring enzymatic activity of culture supernatants after 24 h of growth. No enzyme activity was detected in culture supernatants indicating that the immobilization is effective and no enzyme is released after fixation. About 200 μg of active enzyme were detected in 25 cm^2^ flask corresponding to a final concentration of 8–10 μg cm^-2^. In these conditions, the *Sso*Pox coating was effective in reducing significantly the production of all tested virulence factors.

## Discussion

Foot ulcers are common in diabetic patients mainly due to arteriopathy and neuropathy. The risk of lower-extremity amputation for patients suffering from diabetes is up to 155 times higher than for non-diabetic people (Lavery et al., 2006; Richard et al., 2011). Diabetic foot ulcers are often polymicrobial, bacteria from various types can maintain a chronic infection on their own, or act synergistically in a pathogenic biofilm to cause infection. *P. aeruginosa* is one of the most frequent pathogens isolated in diabetic foot ulcers, particularly in warmer countries (Asia and Africa) where Gram-negative bacilli are more prevalent (Rastogi et al., 2016). This bacterium has developed many strategies to counteract antimicrobial treatments such as antibiotics (Hancock and Speert, 2000). Biofilm formation (Whiteley et al., 2001), β-lactamases (Lauretti et al., 1999) and increased efflux rates (Poole et al., 1993) are part of the molecular arsenal that *P. aeruginosa* deploys to resist anti-bacterial agents, resulting in severe economic and health outcomes (Carmeli et al., 1999). The quest for new therapeutic strategies to fight *P. aeruginosa* infections is highly challenging.

Remarkably, among the 51 strains assayed in this study, the clinical isolates of *P. aeruginosa* harbored distinct phenotypes with only 16 strains producing the three virulence factors as the model strains PAO1 and PA14. Most strains produced only one or two of the studied virulence factors in the conditions tested. In addition, the levels of each virulence factor were highly variable from one isolate to another. Such phenotypic variations have already been reported and underline the necessity to test potential therapeutic molecules on clinical isolates (Grosso-Becerra et al., 2014).

In this study, the ability of the lactonase variant *Sso*Pox-W263I to decrease virulence in clinical isolates of *P. aeruginosa* was evaluated. This variant was selected for an increased catalytic efficiency toward 3-oxo-C_12_ AHL (45-fold increase as compared to the wild-type enzyme) while maintaining a high thermostability (*T*m = 88°C). Three virulence factors were assayed: pyocyanin production, protease secretion and biofilm formation. *Sso*Pox-W263I was shown to drastically affect the synthesis of both virulence factors and biofilm while the QSI C-30 and 5-FU only showed moderate abilities, at their optimum dose, in the same conditions. These results were obtained *in vitro* using a rich medium in which bacterial growth is fast and without pre-treatment of the cultures with the QSI which could explain their poor activity. Notably, the use of 5-FU on PAO1 cultures led to a reduced growth rate of bacteria, possibly indicating some level of toxicity.

Even though *Sso*Pox targets, in principle, only one QS system used by *P. aeruginosa* its QQ potential was very effective. Proteolytic activity was decreased by at least half in 67% of the strains, pyocyanin production in 68% and biofilm in 82%. The use of lactonases as biocontrol agents in bacterial infection therefore seems very promising. Moreover, upon lactonase treatment, no increase in proteolytic activity or pyocyanin production was noticed, only biofilm formation of one strain was slightly enhanced upon treatment with *Sso*Pox but was not statistically relevant. Conversely, the increase of virulence factor production after treatment with QSI molecules has already been reported, when clinical strains of *P. aeruginosa* isolated from cystic fibrosis patients were treated with C-30 and 5-FU and when isolates from diverse origins (urinary tract, wound infection, blood and respiratory tract) were exposed to natural inhibitors such as catechin, caffeine, curcumin and salicylic acid (García-Contreras et al., 2013, 2015; Sethupathy et al., 2016). Similar observations are made in this study with several tested isolates after treatment with C-30 or 5-FU but never after treatment with *Sso*Pox, showing that *Sso*Pox does not induce any deleterious effect *in vitro* as compared to the QSI under the conditions used here.

Resistance of *P. aeruginosa* clinical isolates to QSI has previously been described (Maeda et al., 2012; García-Contreras et al., 2013, 2015). The reported resistance mechanisms involved increased activity of antibiotic efflux pumps to move C-30 more efficiently out of the bacterial cell. Such mutations were obtained *in vitro* by evolving a model strain in the presence of C-30. They were also identified in natural isolates that had never been challenged with C-30. Other reported mutations induce a reduced uptake of C-30 (García-Contreras, 2016). Nevertheless such mutant strains would not show any resistance to *Sso*Pox quenching potential as the enzyme neither enters the bacteria nor binds to a cellular receptor. Even though no growth delay was observed using *Sso*Pox, suggesting no or very low selection pressure, other resistance mechanisms could appear. Bacteria could increase AHL production, produce an enzyme inhibitor, or modify the AHL molecule to prevent its recognition by the enzyme. However, AHL overproduction would represent a significant metabolic cost that would decrease the bacterial fitness in non-selective environments. Modifying the AHL molecules would involve several mutations, first at the level of the synthase then at the level of the receptor. Furthermore, such resistance mechanisms could be easily circumvented by increasing the amount of enzyme or by broadening its spectrum to target more diverse AHL molecules.

Lastly, *Sso*Pox was successfully immobilized in the cultivation flasks using polyurethane and glutaraldehyde. Under these conditions the enzyme was still active and QQ was achieved with a reduction of each virulence factor tested. In addition, no release of the enzyme was measured. Other enzymes have already been described and successfully used to quench *P. aeruginosa*. For example acylases from *Pectobacterium atrosepticum* and *Agrobacterium tumefaciens* have been used to degrade AHL from *P. aeruginosa* (Sunder et al., 2016). Even though the acylases efficiently decreased PAO1 virulence factor production, the stability of these enzymes at high temperatures (above 60°C) was really poor offering a limited industrial potential. The acylase from *Aspergillus meleus* was immobilized in coatings and decreased *P. aeruginosa* biofilm formation (Ivanova et al., 2015; Grover et al., 2016). Nevertheless these reports do not mention the industrial potential of such enzymes regarding their stability and tolerance to solvents, temperature, and bacterial secretions. Regarding the use of lactonases as QQ agents, MomL, a lactonase produced by the marine bacterium *Muricauda olearia* was showed to degrade AHL *in vitro* and to decrease virulence of *P. aeruginosa* PAO1 (Tang et al., 2015). To our knowledge, unlike QSI no QQ enzyme was tested on a wide range of clinical isolates but only on model strains which behaviors differ from natural isolates. The biochemical characteristics of *Sso*Pox-W263I have already been assessed and reported (Hiblot et al., 2013; Rémy et al., 2016b). The data presented here, together with the high resistance of *Sso*Pox and its potential to meet industrial constraints, open the way to incorporating *Sso*Pox in medical devices such as functionalised catheters and anti-virulence dressings. To fully assess the quenching potential of *Sso*Pox and its effect on virulence, infection models should be used.

## Author Contributions

LP, DD, J-PL, ME, and EC designed the study. AG, LP, JB, PJ, and BR performed the experiments. JB, LP, and DD analyzed the data. AG, J-PL, LP, DD, and EC wrote the manuscript.

## Conflict of Interest Statement

ME and EC have a patent WO2014167140 A1 licensed to Gene&GreenTK. LP, DD, ME, and EC report personal fees from Gene&GreenTK during the conduct of the study. All the other authors declare that the research was conducted in the absence of any commercial or financial relationships that could be construed as a potential conflict of interest. The reviewer GR and handling Editor declared their shared affiliation and the handling Editor states that the process nevertheless met the standards of a fair and objective review.
