# Supplementary material for: Effect of Quorum Quenching Lactonase in Clinical Isolates of Pseudomonas aeruginosa and Comparison with Quorum Sensing Inhibitors
Source: Front Microbiol. 2017 Feb 14;8:227. doi: 10.3389/fmicb.2017.00227 (PMC5306132; doi:10.3389/fmicb.2017.00227)
Supplement: Supplementary file 1 [file Data_Sheet_1.DOCX]

**A**

**C**

**B**

**D**

**Supplementary Figure 1. Dose response of *Sso*Pox with the model strains PAO1 and PA14.**

Three *Sso*Pox concentrations (0.1 mg ml^-1^, 0.5 mg ml^-1^, 1.0 mg ml^-1^) were used. Values represent the mean ratios between treated and untreated samples of three experiments. The effect of the QQ enzyme on growth, pyocyanin secretion, protease activity and biofilm formation was evaluated. Stars indicate a *p-value*<0.05 according to Student's t-test.

**B**

**A**

**C**

**D**

**Supplementary Figure 2. Dose response of 5-FU with the model strains PAO1 and PA14.**

Four 5-FU concentrations (10 µM, 30 µM, 60 µM, 150 µM) were used. Values represent the mean ratios between treated and untreated samples of three experiments. The effect of QSI on growth, pyocyanin secretion, protease activity and biofilm formation was evaluated. Stars indicate a *p-value*<0.05 according to Student's t-test.

**A**

**B**

**C**

**D**

**Supplementary Figure 3. Dose response of C-30 with the model strains PAO1 and PA14.**

Four C-30 concentrations (10 µM, 30 µM, 60 µM, 150 µM) were used. Values represent the mean ratios between treated and untreated samples of three experiments. The effect of QSI on growth, pyocyanin secretion, protease activity and biofilm formation was evaluated. Stars indicate a *p-value*<0.05 according to Student's t-test.

**Supplementary Figure 4. SDS-page of SsoPox-W263I obtained after gel filtration purification**


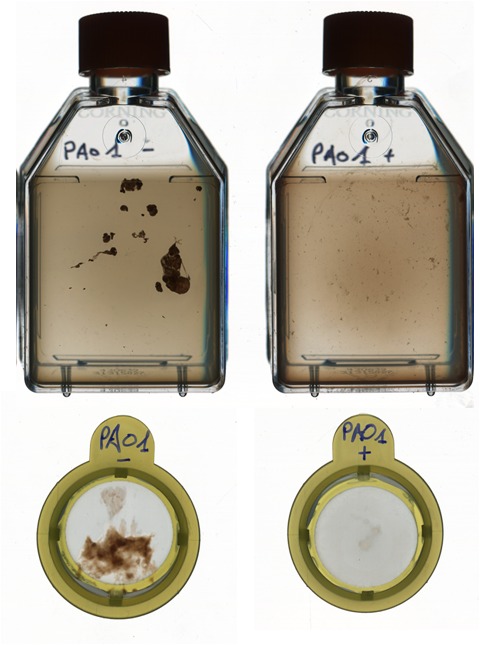


**Supplementary Figure 5. Direct measurement of biofilm by direct weighing of cell aggregates.**

After 48 h of growth, cells were filtered through a 100 µm cell strainer to separate planktonic cells from biofilms. Biofilms were directly weighed in the cell strainers. Left picture represents a culture without *Sso*Pox while right picture represents a culture treated with the enzyme.

**Supplementary Figure 6. Impact of QQ enzyme and QSI on bacterial growth**

The impact of *Sso*Pox (A), C30 (B) and 5-FU (C) on bacterial growth of PAO1 and PA14 for different concentrations.
